# Supplementary material for: Temporal dedifferentiation of neural states with age during naturalistic viewing
Source: Commun Biol. 2025 Sep 30;8:1390. doi: 10.1038/s42003-025-08792-4 (PMC12484978; doi:10.1038/s42003-025-08792-4)
Supplement: Supplementary file 1 — Supplementary Materials [file 42003_2025_8792_MOESM1_ESM.pdf]

Temporal dedifferentiation of neural states with age during naturalistic viewing

Selma Lugtmeijer, Djamari Oetringer, Linda Geerligs‡, Karen L Campbell‡
‡shared senior author

Supplementary Materials

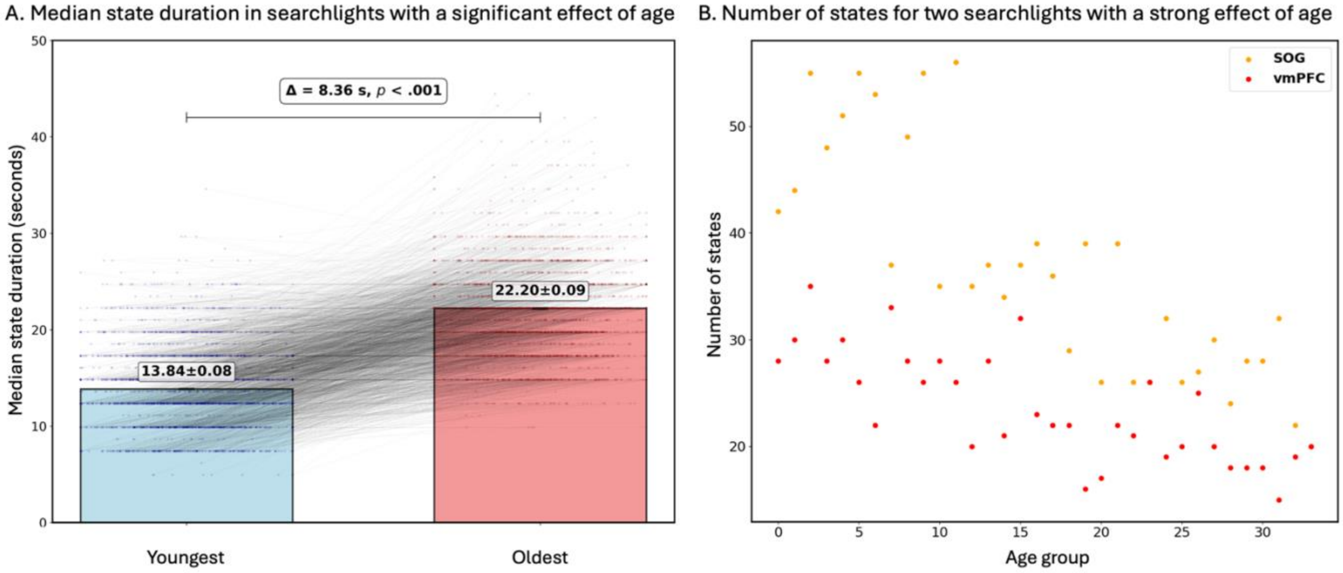

Supplementary figure 1 A) Comparison of median state duration between the youngest group (left) and oldest group (right) for all 2816 significant searchlights that showed an effect of age. Bars show means ± SEM, dots show individual searchlights. Mean Δ = 8.36 seconds, $p < .001$ B) The effect of age group on number of neural states for the vmPFC and SOG.

Supplementary figure 1 shows in panel A for all searchlights with a significant effect of age (after FDR correction) how the median state duration changed with age from the youngest group to the oldest group. The mean change is an increase of 8.36 seconds in state duration (SD = 5.02, range -9.88 – 32.11). 93.9% of significant regions showed longer durations in older adults, in the remaining 6.1% the difference between the youngest and oldest group was either neutral (3.1%) or negative (3.0%) but across all groups all significant correlations were positive. How the number of states can fluctuate across age despite a negative correlation is illustrated in panel B. This gives an example of how the number of neural states changes with increasing age for two searchlights that showed the strongest effect of age. The number of neural states is the inverse of the duration of neural states as both are based on the neural state boundaries estimated by GSBS. In the main manuscript, the median duration between the neural state boundaries is used as measure of interest. Panel B shows the count of the neural states for illustrative purposes.

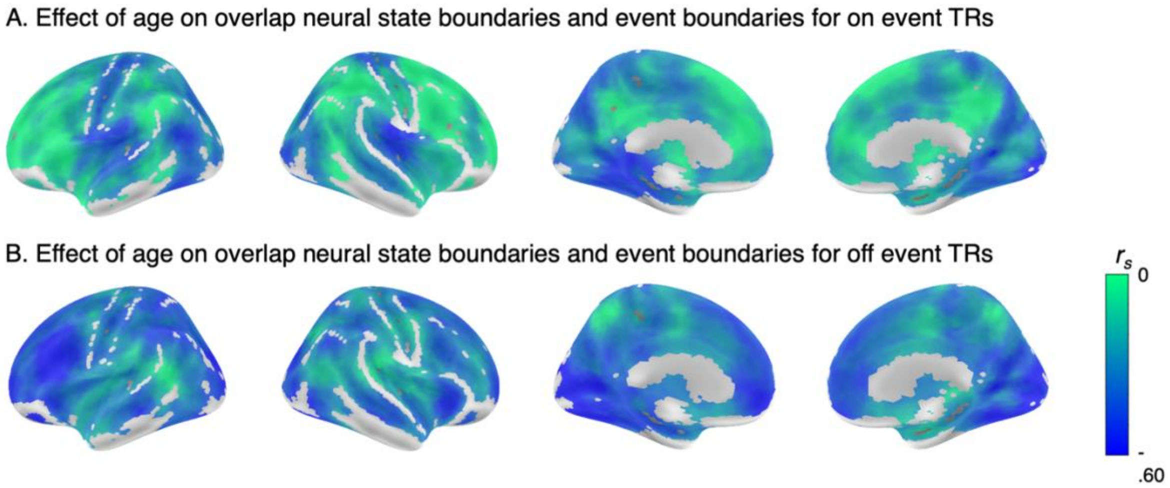

Supplementary figure 2 The effect of age on boundary occurrence for TRs that overlap with perceived events (A) and TRs that do not (B).

Supplementary figure 2 visualizes the difference between alignment and non-alignment with event boundaries for the correlation between age and neural boundary occurrence. In both on event and off event TRs we see an overall decrease in boundary occurrence with age (i.e., longer states), however this effect is stronger for off event TRs (mean correlation across all searchlights $r_{s} =$ -0.36) than event TRs ($r_{s} =$ -0.21). This explains why we observe longer neural states with increasing age without a decrease in overlap between event and neural state boundaries.

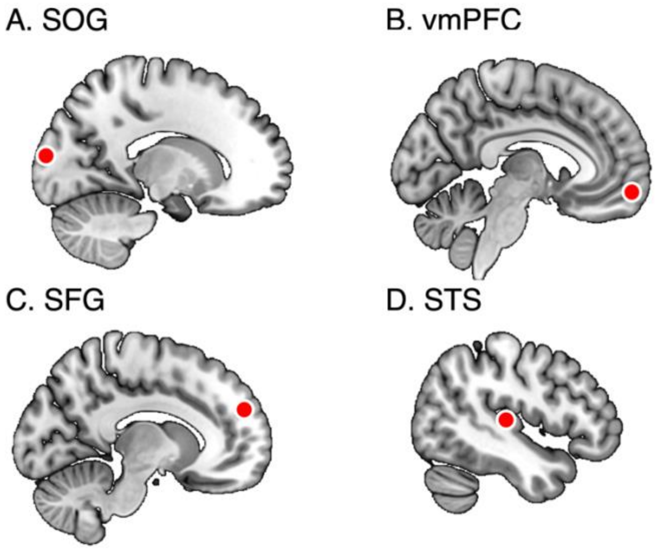

Supplementary figure 3 Location of selected searchlights for single subject GSBS and simulations. Top row: searchlights with a strong effect of age on neural state duration. A. Superior occipital gyrus, SOG, -15 x -96 x 14, SL 1874; B. vmPFC, -6 x 57 x -13, SL 2466. Bottom row: searchlights with a high overlap between perceived event boundaries and neural state boundaries. C. Superior frontal gyrus, SFG, -9 x 51 x 26, SL 2463; D. superior temporal gyrus, STS, -45 x -24 x 6, SL 692. Coordinates in MNI space.

Supplementary figure 3 visualizes the location of the four searchlights that are used in searchlight specific analyses. The two searchlights in the top row are also visualised in the main manuscript in the section *Increase in neural state duration with age*, Figure 2. Those are the searchlights with the highest correlation between age and median state duration. The searchlights on the bottom row are those with a high overlap between perceived event boundaries and neural state boundaries. All four were used for single-subject GSBS to investigate the overlap between neural states and events on a single-subject level.

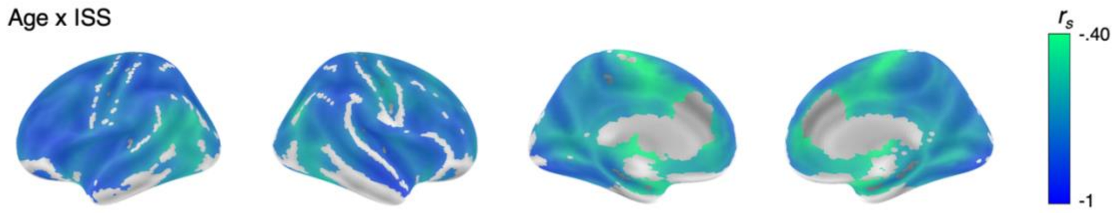

Supplementary figure 4 The effect of age on ISS. Age has a negative effect on ISS across the cortex indicating that with increasing age the neural signal is less similar.

Supplementary figure 4 shows the effect of age on intersubject synchrony (ISS). Age had the strongest negative effect on ISS bilaterally in the temporal pole, the dorsolateral PFC, and the inferior parietal lobe. With increasing age, the neural signal is less similar. This spatial pattern of correlations only had a small overlap with regions where the effect of age on neural state duration was strongest (r_s = -.09, p < .001, Figure 2 main manuscript), indicating that age affects ISS and state duration largely in different regions of the brain. This suggests that there is a different driving force behind the age-related differences in neural state duration and the differences in ISS.

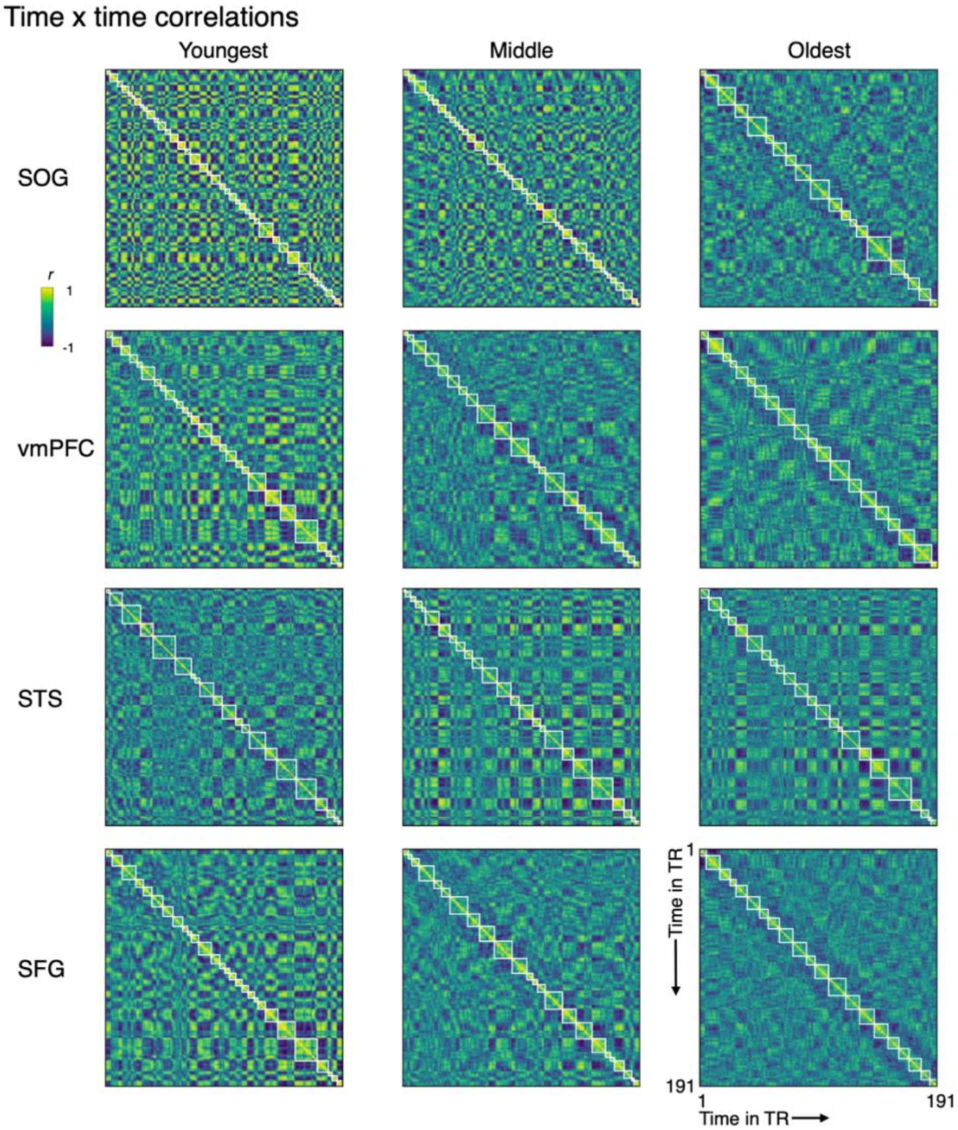

Supplementary figure 5 Time-by-time matrices from the youngest, middle, and oldest group for four selected searchlights as reference for effects of age.

Supplementary figure 5 shows the time-by-time matrices for the four selected searchlights for the youngest, middle, and oldest group. These are based on the actual data and can be used as a reference for the simulation results.

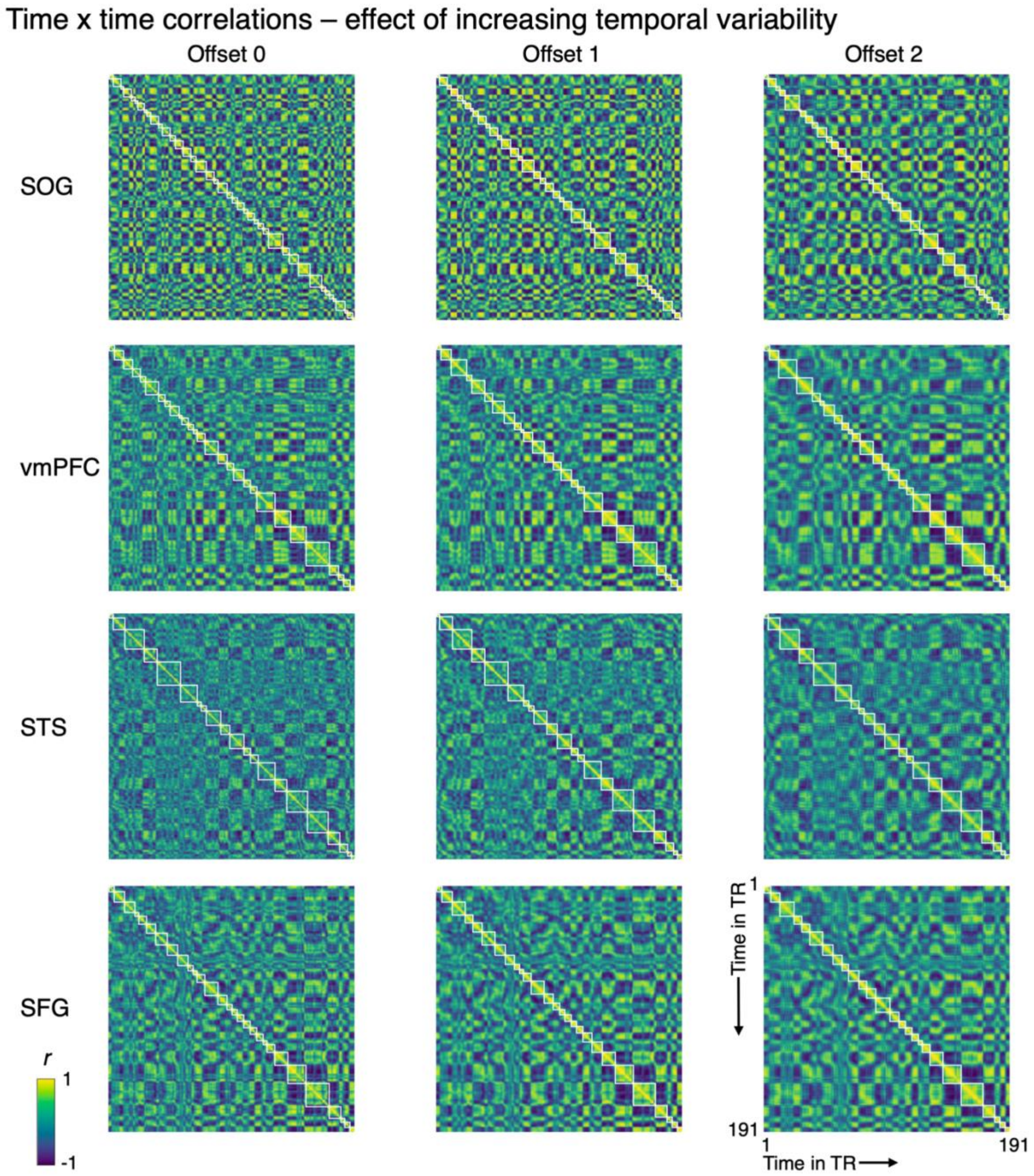

Supplementary figure 6 The effect of increasing temporal variability with 1 or 2 TRs relative to the data of the youngest subgroup, visualized in time-by-time matrices for four selected searchlights, two that show a strong effect of overlap (top rows) and two with strong effect of age on duration (bottom rows).

Supplementary figures 6 till 8 show results of simulations. Supplementary figure 6 visualises how interindividual temporal variability in the occurrence of state boundaries affected the duration of neural states by shifting neural state boundaries of the youngest group by one or two TRs. As can be seen by comparing supplementary figure 6 with supplementary figure 5, the time-by-time matrices based on simulations look dissimilar from those of older adults.

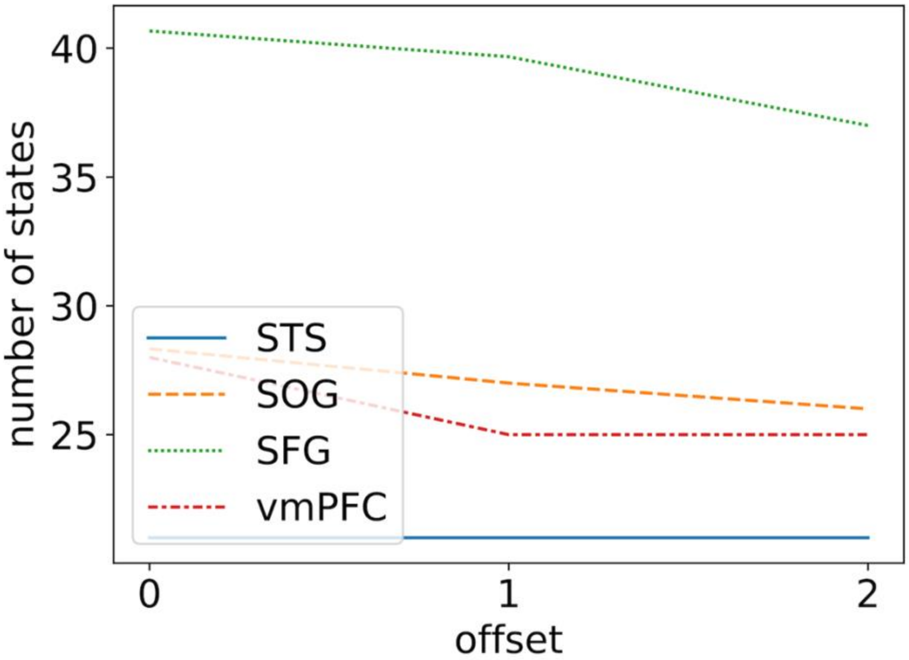

Supplementary figure 7 The effect of increasing temporal variability on the number of states for four selected searchlights.

Supplementary figures 7 shows that the number of states tend to decrease slightly with a simulated increase in inter-individual variability. Importantly, these effects are not in the range that we observe with aging. While with advancing age, the number of states can decline very steeply (e.g. from 41 to 25 states in the SOG), the simulations show a much smaller decrease (largest difference is from 41 to 37), suggesting that increased variability in the timing of state boundaries cannot explain the observed increase in neural state durations with age.

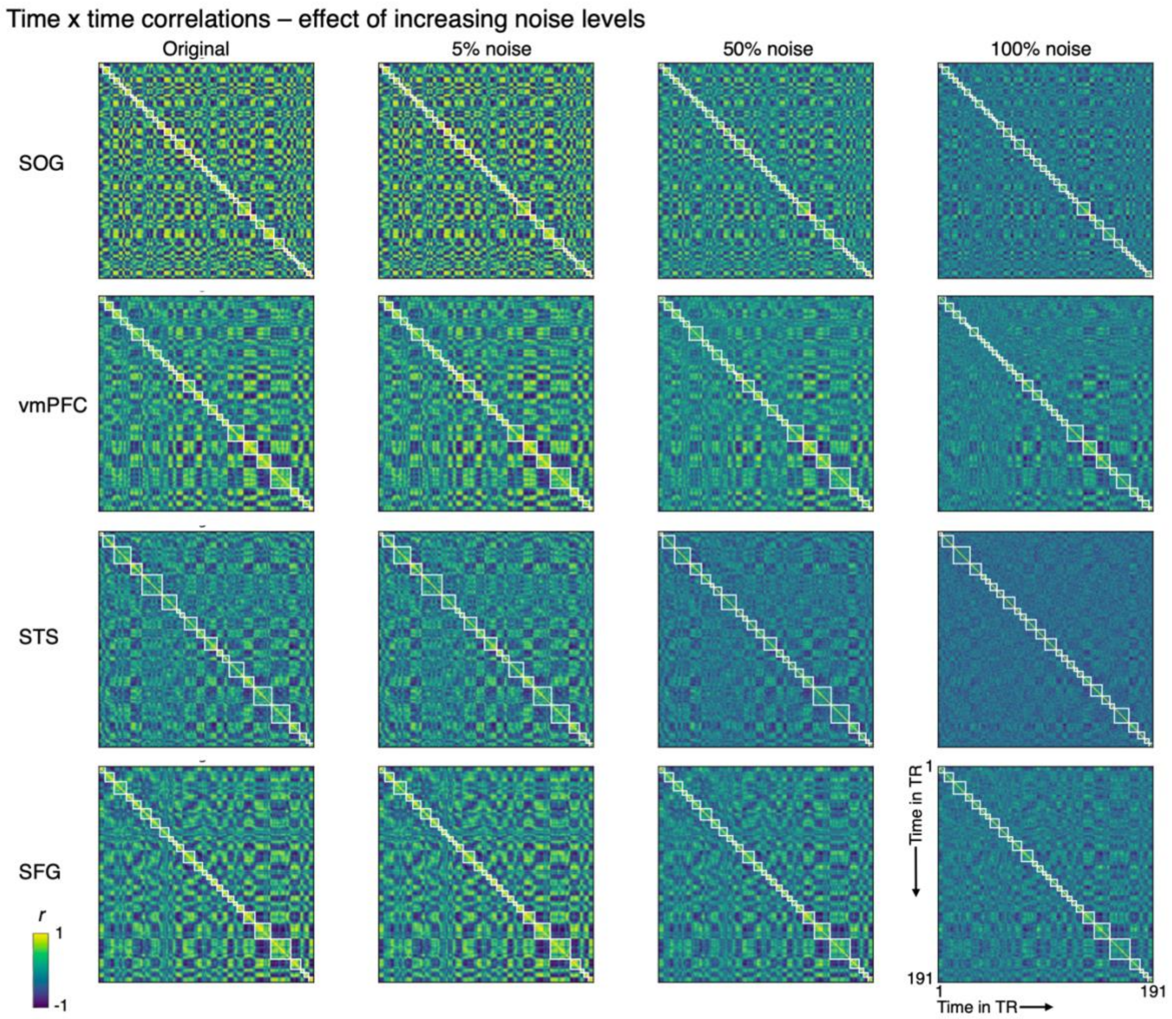

Supplementary figure 8 The effect of increasing random noise relative to the data of the youngest group, visualized in time-by-time matrices for four selected searchlights.

In the next simulation, we investigated whether decreased signal-to-noise levels could explain the observed increase in neural state durations with age. Supplementary figure 8 visualises the effect of adding increasing levels of random noise to the data of the youngest group to investigate if this could explain the increase in neural state durations with age. While visually this made the matrices look more similar to those of older adults (see supplementary figure 5), we did not observe a systematic decrease in the number of neural states with higher levels of noise (supplementary figure 9).

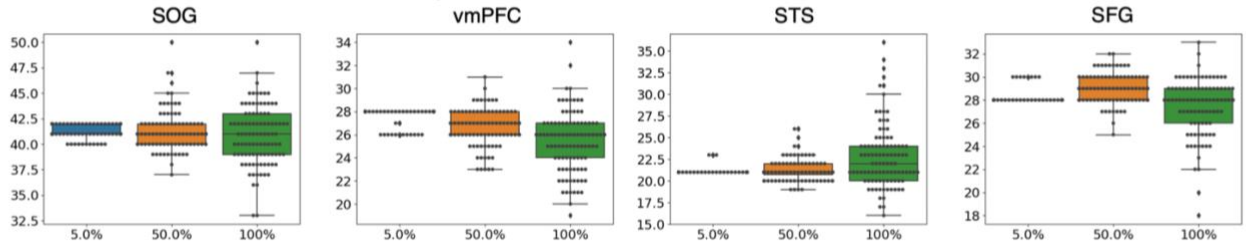

Supplementary figure 9 The effect of increasing noise levels on the number of states for four selected searchlights.

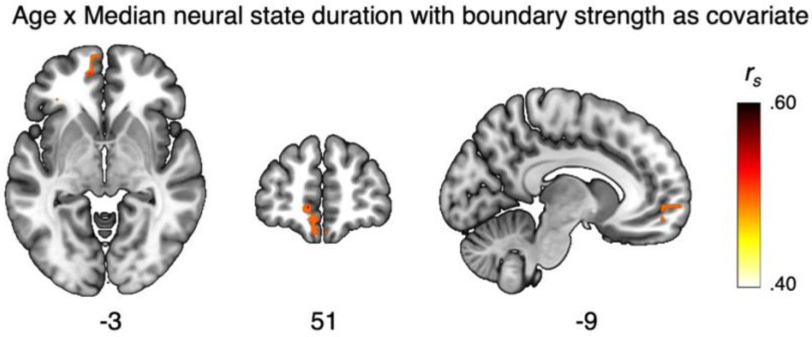

Supplementary figure 10 The correlation between age and state duration with boundary strength as covariate (FDR corrected). Correlations between .49 and .59. Color scale has a wider range to ensure clearly visible results.

Our results show that neural state boundaries were weaker in older adults which might explain part of the observed lengthening of neural states with age. Supplementary figure 10 shows that the effect of age on state duration persisted in the vmPFC whilst taking boundary strength into account. This suggests that the lengthening of neural states cannot be fully explained by the weakening of neural states with age.

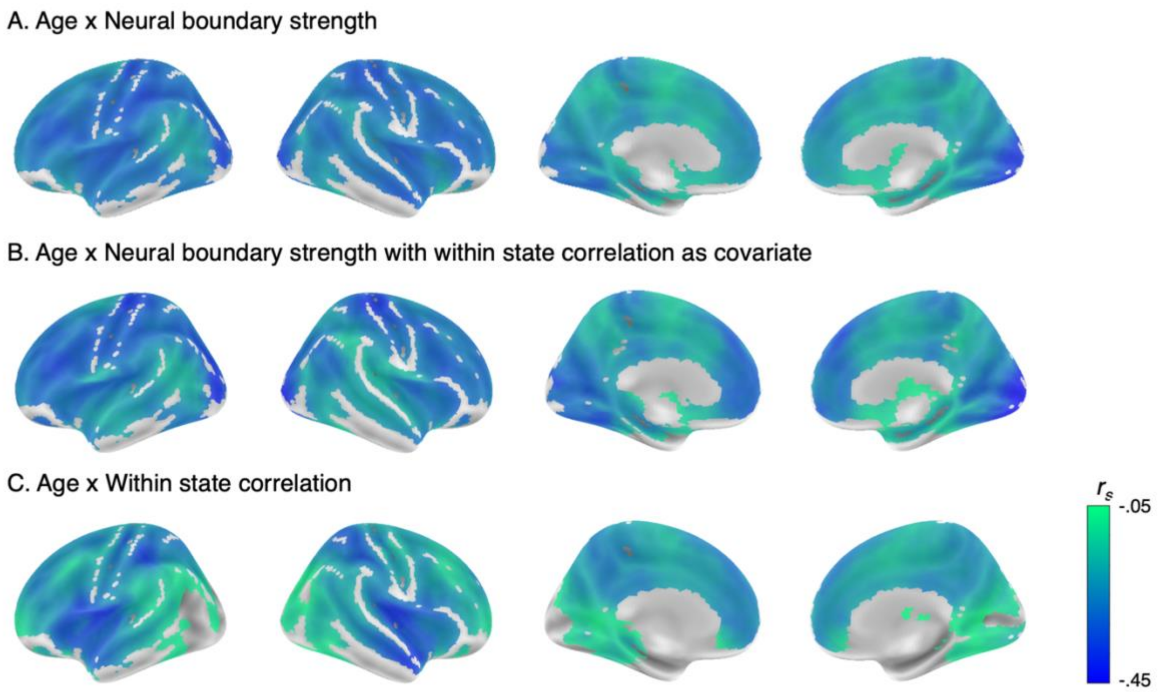

Supplementary figure 11 The effect of age on neural state boundary strength and within state correlation. A) The effect of age on boundary strength, a replication of the original analysis reported in the section *Weaker neural state boundaries only partly explain the effect of age on state duration,* Figure 7. B) The effect of age on boundary strength adjusted for within state correlations. C) Correlation between within state correlations and age.

Supplementary Analysis 1 Model comparison for age relationships with state duration and boundary strength

In the main manuscript, we examined all relationships with age using Spearman's rank correlation coefficients. This non-parametric method is advantageous as it detects monotonic trends without assuming a specific functional form and accommodates categorical variables, in our case age groups.

Here, we present supplementary analyses aimed at identifying the optimal functional form of the age–neural state relationship. Specifically, we assessed the relationship between age groups and median state duration, and between continuous age and neural boundary strength. We compared four models—linear, quadratic, exponential, and logarithmic—using the Akaike Information Criterion (AIC) for model selection. For each searchlight, all four models were fit using curve fitting, and the model with the lowest AIC was selected, balancing goodness-of-fit with model complexity.

For the relationship between age group and median state duration, linear models provided the best fit in 60.5% of searchlights, followed by quadratic (21.8%), exponential (15.6%), and logarithmic (2.1%) models. This suggests that the relationship between age and state duration is predominantly linear across the brain.

In contrast, for the relationship between continuous age and boundary strength, quadratic models were optimal in 72.8% of searchlights, followed by linear (25.8%), logarithmic (0.9%), and exponential (0.5%) models. The AIC differences between quadratic and linear models indicate nonlinear age-related effects on boundary strength (see supplementary figure 12).

In line with the results in our main manuscript, these additional analyses suggest that aging has a different effect on neural state duration than on boundary strength. Whereas state duration increases linearly in large parts of the brain with age, boundary strength follows a quadratic shape with the highest strength in the middle of adulthood but still a lower tail in older age compared to younger age. To illustrate this, the figure below shows the relationship between age and boundary strength for the six searchlights with the biggest AIC difference between a linear and quadratic fit.

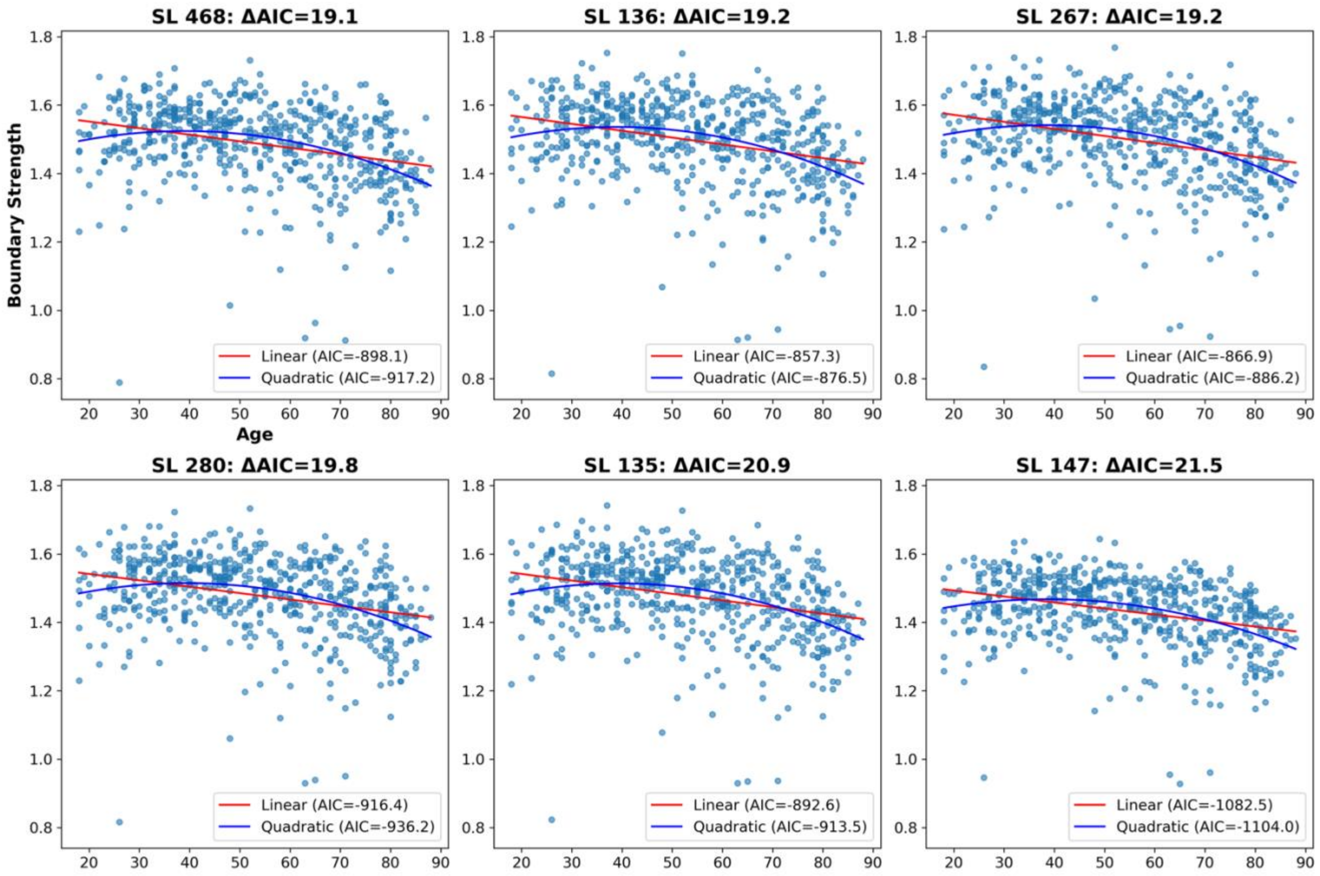

Supplementary figure 12 Age - boundary strength relationships for six searchlights with the largest AIC differences favoring a quadratic over a linear fit. Each panel shows individual data points for all searchlights, with linear (blue) and quadratic (red) trend lines overlaid.

Supplementary analysis 2 Network involvement

Weaker boundaries might result from decreased similarity within neural states (resulting in a less coherent neural pattern per state or more noisy data) as well as from decreased distinction of neural patterns across states. Therefore, we also tested whether the correlation between age and boundary strength remained after correcting for average within state correlation (as reduced within-state coherence could give the impression that boundary strength decreases with age). However, the effect of age on boundary strength remained, suggesting that weaker state boundaries with age cannot be explain by a lack of coherence within states. Although it should be noted that within-state correlations also declined with age.

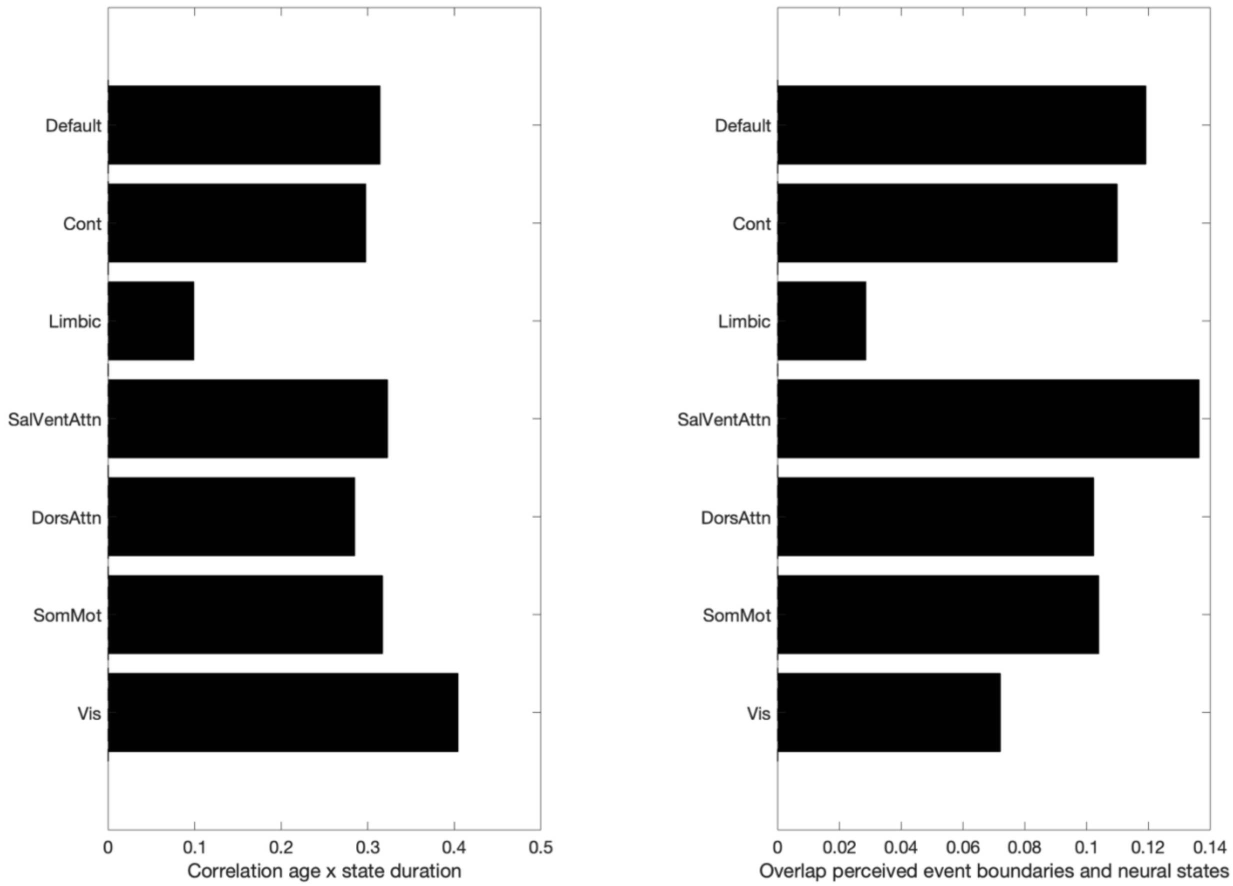

Supplementary figure 13 Network involvement in the correlation between age group and median neural state duration on the left and overlap between perceived event boundaries and neural states on the right.

Our results suggest that a different set brain areas is involved in event boundary detection than those most affected by age. Here we investigated network-involvement of those effects using the Schaefer 7-network 200 parcellation by comparing the strength of effects per network (supplementary figure 13). Age-related increases in neural state duration were most pronounced in the visual network, whilst the correspondence between perceived event boundaries and neural state boundaries was highest in the salience/ventral attention network.

Supplementary table 1 Age group descriptives

Group	*M* age	min	max	*M* qualification[1]	sex (N men)
1	19.75	18	23	3.13	6
2	24.24	23	25	3.35	4
3	26.71	26	28	3.71	6
4	28.47	28	29	3.94	7
5	30.59	29	32	3.59	9
6	32.65	32	34	3.65	5
7	34.41	34	35	3.71	8
8	36.12	35	37	3.82	12
9	37.53	37	39	3.81	13
10	39.59	39	40	3.82	10
11	41.12	40	42	3.47	9
12	43.24	42	44	3.65	6
13	45.29	44	46	3.24	10
14	46.76	46	47	3.47	7
15	48.12	47	49	3.59	6
16	50.06	49	51	3.71	8
17	51.88	51	53	3.41	7
18	53.88	53	55	3.29	8
19	55.71	55	57	3.18	8
20	57.71	57	59	3.65	7
21	59.71	59	60	3.18	9
22	61.59	61	63	3.35	6
23	63.47	63	64	3.47	5
24	65.35	64	66	3.53	11
25	67.41	66	68	3.35	12
26	69.00	68	70	2.63	12
27	70.82	70	72	3.29	13
28	72.71	72	74	3.06	9
29	75.47	74	76	3.06	9
30	77.65	77	78	3.00	7
31	78.94	78	79	3.29	8
32	80.18	80	81	3.53	10
33	82.12	81	83	2.71	9
34	85.18	83	88	2.88	7

Note: [1]qualification measured in four levels; 1 = none > 16, 2 = GCSE grade, 3 = A' levels, 4 = university
