# Supplementary material for: Temporal dedifferentiation of neural states with age during naturalistic viewing
Source: Commun Biol. 2025 Sep 30;8:1390. doi: 10.1038/s42003-025-08792-4 (PMC12484978; doi:10.1038/s42003-025-08792-4)
Supplement: Supplementary file 2 — Reporting Summary [file 42003_2025_8792_MOESM2_ESM.pdf]

nature portfolio

Corresponding author(s): Lugtmeijer, Selma

Last updated by author(s): 14/8/25

Reporting Summary

Nature Portfolio wishes to improve the reproducibility of the work that we publish. This form provides structure for consistency and transparency in reporting. For further information on Nature Portfolio policies, see our Editorial Policies and the Editorial Policy Checklist.

Statistics

For all statistical analyses, confirm that the following items are present in the figure legend, table legend, main text, or Methods section.

n/a	Confirmed	
☐	☒	The exact sample size (*n*) for each experimental group/condition, given as a discrete number and unit of measurement
☐	☒	A statement on whether measurements were taken from distinct samples or whether the same sample was measured repeatedly
☐	☒	The statistical test(s) used AND whether they are one- or two-sided<br>*Only common tests should be described solely by name; describe more complex techniques in the Methods section.*
☐	☒	A description of all covariates tested
☐	☒	A description of any assumptions or corrections, such as tests of normality and adjustment for multiple comparisons
☐	☒	A full description of the statistical parameters including central tendency (e.g. means) or other basic estimates (e.g. regression coefficient) AND variation (e.g. standard deviation) or associated estimates of uncertainty (e.g. confidence intervals)
☐	☒	For null hypothesis testing, the test statistic (e.g. *F*, *t*, *r*) with confidence intervals, effect sizes, degrees of freedom and *P* value noted<br>*Give P values as exact values whenever suitable.*
☒	☐	For Bayesian analysis, information on the choice of priors and Markov chain Monte Carlo settings
☒	☐	For hierarchical and complex designs, identification of the appropriate level for tests and full reporting of outcomes
☐	☒	Estimates of effect sizes (e.g. Cohen's *d*, Pearson's *r*), indicating how they were calculated

Our web collection on statistics for biologists contains articles on many of the points above.

Software and code

Policy information about availability of computer code

Data collection	NA
Data analysis	The code used to generate the results in this paper is available at https://osf.io/4z67t/ and https://github.com/slugtmeijer and additional code at https://github.com/lgeerligs. The GSBS algorithm used is released in a Python package: https://pypi.org/project/statesegmentation/

For manuscripts utilizing custom algorithms or software that are central to the research but not yet described in published literature, software must be made available to editors and reviewers. We strongly encourage code deposition in a community repository (e.g. GitHub). See the Nature Portfolio guidelines for submitting code & software for further information.

Data

Policy information about availability of data

All manuscripts must include a data availability statement. This statement should provide the following information, where applicable:

- Accession codes, unique identifiers, or web links for publicly available datasets
- A description of any restrictions on data availability
- For clinical datasets or third party data, please ensure that the statement adheres to our policy

The data used in this project can be requested via - https://camcan-archive.mrc-cbu.cam.ac.uk/dataaccess/

Research involving human participants, their data, or biological material

Policy information about studies with human participants or human data. See also policy information about sex, gender (identity/presentation), and sexual orientation and race, ethnicity and racism.

Reporting on sex and gender	Gender (self-identified) has been collected as described in the published protocol of the Cam-CAN study. In our study sample of 577 adults, 293 identified as female. In non of the analyses gender was taken into account as analyses were conducted at group level to study age effects.
Reporting on race, ethnicity, or other socially relevant groupings	Not applicable
Population characteristics	The only relevant participant characteristic included is age. For most analyses groups of participants sorted in age bins were used. Only for one set of analyses age was used as a continuous variable.
Recruitment	This study reports on data from 577 adults (293 females) who were aged 18 - 88 (mean age 53.39, SD = 18.42) from the healthy, population-based cohort tested in stage II of the Cam-CAN project (Shafto et al., 2014; Taylor et al., 2017).
Ethics oversight	Cambridgeshire 2 (now East of England – Cambridge Central) Research Ethics Committee

Note that full information on the approval of the study protocol must also be provided in the manuscript.

Field-specific reporting

Please select the one below that is the best fit for your research. If you are not sure, read the appropriate sections before making your selection.

☐ Life sciences ☒ Behavioural & social sciences ☐ Ecological, evolutionary & environmental sciences

For a reference copy of the document with all sections, see nature.com/documents/nr-reporting-summary-flat.pdf

Behavioural & social sciences study design

All studies must disclose on these points even when the disclosure is negative.

Study description	It's a quantitative cross sectional study
Research sample	This study reports on data from 577 adults (293 females) who were aged 18 - 88 (mean age 53.39, SD = 18.42) from the healthy, population-based cohort tested in stage II of the Cam-CAN project (Shafto et al., 2014; Taylor et al., 2017). This is a representative cohort with even spread of adult ages, ideal for a lifespan study.
Sampling strategy	We made use of the already available Cam-CAN study, which is a large population based cohort.
Data collection	The full study protocol has been published (Shafto et al., 2014; Taylor et al., 2017). Date collection for this specific study is that of a movie fMRI task.
Timing	2011-2014
Data exclusions	Participants had English as a first language, normal or corrected-to-normal vision and hearing, no contraindications to MRI, and no neurological disorders.
Non-participation	For 30 participants, there was either a problem with running the ME-ICA denoising or a problem with the normalization; these participants were not included in further analyses. Furthermore, participants with a high level of mean head motion (2 SDs above the mean, 32 participants) and participants for whom nearly all components were removed during the ME-ICA denoising (>88% of all components, 2 participants) were not included in the analyses.
Randomization	Participants were split into 34 age groups of the same group size. No characteristics were taken into account. Gender split within each group is reported in supplementary materials.

Reporting for specific materials, systems and methods

We require information from authors about some types of materials, experimental systems and methods used in many studies. Here, indicate whether each material, system or method listed is relevant to your study. If you are not sure if a list item applies to your research, read the appropriate section before selecting a response.

Materials & experimental systems

n/a	Involved in the study
☒	☐ Antibodies
☒	☐ Eukaryotic cell lines
☒	☐ Palaeontology and archaeology
☒	☐ Animals and other organisms
☒	☐ Clinical data
☒	☐ Dual use research of concern
☒	☐ Plants

Methods

n/a	Involved in the study
☒	☐ ChIP-seq
☒	☐ Flow cytometry
☐	☒ MRI-based neuroimaging

Plants

Seed stocks

Report on the source of all seed stocks or other plant material used. If applicable, state the seed stock centre and catalogue number. If plant specimens were collected from the field, describe the collection location, date and sampling procedures.

Novel plant genotypes

Describe the methods by which all novel plant genotypes were produced. This includes those generated by transgenic approaches, gene editing, chemical/radiation-based mutagenesis and hybridization. For transgenic lines, describe the transformation method, the number of independent lines analyzed and the generation upon which experiments were performed. For gene-edited lines, describe the editor used, the endogenous sequence targeted for editing, the targeting guide RNA sequence (if applicable) and how the editor was applied.

Authentication

Describe any authentication procedures for each seed stock used or novel genotype generated. Describe any experiments used to assess the effect of a mutation and, where applicable, how potential secondary effects (e.g. second site T-DNA insertions, mosiacism, off-target gene editing) were examined.

Magnetic resonance imaging

Experimental design

Design type

movie fMRI

Design specifications

8-min movie

Behavioral performance measures

no behavioral outcome measures

Acquisition

Imaging type(s)

functional

Field strength

3

Sequence & imaging parameters

For the fMRI sequence, 193 volumes of movie data were acquired with a 32-channel head-coil, using a multi-echo, T2 ? -weighted echo-planar imaging (EPI) sequence. Each volume contained 32 axial slices (acquired in descending order), with slice thickness of 3.7 mm and interslice gap of 20% (repetition time (TR) = 2470 ms; five echoes [TE = 9.4 ms, 21.2 ms, 33 ms, 45 ms, 57 ms]; flip angle = 78°; field-of- view = 192 ×192 mm; voxel-size = 3 ×3 ×4.44 mm), the acquisition time was 8 min and 13 s. A high-resolution (1 mm isotropic) T1 - weighted image was additionally acquired.

Area of acquisition

Whole brain

Diffusion MRI ☐ Used ☒ Not used

Preprocessing

Preprocessing software

Analysis of functional neuroimages software (AFNI; version AFNI_17.1.01; https://afni.nimh.nih.gov) and the statistical parametric mapping software (SPM12; (http://www.fil.ion.ucl.ac.uk/spm) were used for preprocessing. The AFNI parts of preprocessing included deobliquing of each TE, slice time correction, realignment of each TE to the first TE in the run, and multi-echo independent component analysis (ME-ICA) denoising.

Normalization

In SPM, the ME-ICA denoised data were co-registered, followed by DARTEL intersubject alignment which allows for transformation to an age-representative template that is subsequently transformed to Montreal Neurological Institute (MNI) space.

Normalization template

DARTEL intersubject alignment which allows for transformation to an age-representative template that is subsequently transformed to Montreal Neurological Institute (MNI) space.

Noise and artifact removal

ME-ICA denoising data - participants with a high level of mean head motion (2 SDs above the mean, 32 participants) and

Noise and artifact removal

participants for whom nearly all components were removed during the ME-ICA denoising (>88% of all components, 2 participants) were not included in the analyses.

Volume censoring

NA

Statistical modeling & inference

Model type and settings

To optimally align voxels across participants of each age group, we used whole-brain searchlight hyperalignment as implemented in the PyMVPA toolbox. Subsequently, Greedy State Boundary Search (GSBS) was used to estimate neural state boundaries. Neural state durations and locations o boundaries were used in analyses.

Effect(s) tested

The first step in our analysis was to investigate whether this life-span sample showed similar patterns of neural state durations as our previous work in a younger sample (Geerligs et al., 2022). To do this, we first computed the metric of interest (i.e., median state duration and variability in state duration) per age group before averaging the data across age-groups to visualize patterns in younger, middle, and older adults (11, 12, and 11 groups, respectively). As the variability in state duration metric tends to increase with median duration, we used a nonparametric version of the coefficient of variation, calculated by dividing the interquartile range (IQR) by median state duration.

Our main aim was to examine the effect of age on neural states. Age effects on median state duration and variability in state duration were estimated with the Spearman's rank correlation coefficient comparing the 34 age groups. Analyses were FDR corrected across searchlights. For visualization of the effect of age on median state duration, searchlights with the strongest effect of age were selected and time-by-time correlations matrices were plotted for the youngest, middle, and oldest groups.

To assess the overlap in neural state boundaries and perceived event boundaries, we used the absolute overlap metric described by Geerligs et al. (2022). The absolute overlap computes the total number of event and neural states boundaries that overlap and subsequently scales this with respect to the maximal and the expected number of overlapping boundaries. A measure of one indicates that all neural state boundaries align with an event boundary, zero indicates an overlap as expected by chance (e.g., if there are 20 neural states boundaries, 10 event boundaries and 100 time points, you would expect 20/100*10/100*100 = 2 boundaries to overlap by chance). To account for the hemodynamic response in the BOLD signal, we added 5 seconds to the onset of each perceived event boundary, and then allowed for a 1-second window around that timing to account for slight variability in alignment with TRs (some perceived event boundaries are at the boundary of two TRs), finally we converted seconds to TRs (i.e., divided by 2.47). To calculate the significance of the overlap, we tested with a ranksum test whether the boundary overlap was significantly different from zero across the 34 age groups after false discovery rate (FDR) correction. To visualize patterns, we averaged that overlap in younger, middle, and older adults (11, 12, and 11 groups, respectively). The effect of age on overlap was estimated with the Spearman's rank correlation coefficient comparing the 34 age groups, FDR corrected across searchlights. Subsequently, using a correlation test, we assessed the spatial overlap between the effects of age on state duration and on neural state boundary correspondence to perceived event boundaries to determine if these effects are observed in the same brain regions.

Finally, we examined if age affected neural state boundary occurrence differently for TRs that overlap with a perceived event boundary versus those that do not. For each TR, boundary occurrence across age groups was calculated. TRs were split into those that overlapped with a perceived event (allowing a 1-second window), and those that were non-event TRs. The effect of age on neural boundary occurrence was estimated with Spearman’s rank correlation coefficients in each of these types of TRs. This was done for the average across all searchlights, as well as for each searchlight separately.

Specify type of analysis: ☒ Whole brain ☐ ROI-based ☐ Both

Statistic type for inference

(See Eklund et al. 2016)

The input to the GSBS algorithm consists of a set of voxel time courses within a searchlight. For the main analyses, spherical searchlights were scanned within a group specific mask with a step size of two voxels and a radius of three voxels. To define the mask, a threshold was set at 70% of the mean activity across all voxels for each participant. Voxels meeting this threshold in all participants were included. The final mask only included voxels overlapping with the grey matter SPM template, thresholded at >0.2 probability. This resulted in 5204 searchlights. Searchlights had an average size of 97 voxels (max: 123; IQR: 82–115); variation in size was a consequence of exclusion of out-of-brain voxels. Searchlights had to have a minimum size of 15 voxels to be included in the analysis.

Correction

Results were FDR corrected across searchlights.

Models & analysis

n/a	Involved in the study
☒	☐ Functional and/or effective connectivity
☒	☐ Graph analysis
☒	☐ Multivariate modeling or predictive analysis
